# Supplementary material for: Computation-based regulation of excitonic effects in donor-acceptor covalent organic frameworks for enhanced photocatalysis
Source: Nat Commun. 2023 May 29;14:3083. doi: 10.1038/s41467-023-38884-w (PMC10227069; doi:10.1038/s41467-023-38884-w)
Supplement: Supplementary file 1 — Supplementary Information [file 41467_2023_38884_MOESM1_ESM.pdf]

# Supplementary Information

## Computation-Based Regulation of Excitonic Effects in Donor-Acceptor Covalent Organic Frameworks for Enhanced Photocatalysis

Yunyang Qian[1,*], Yulan Han[2,*], Xiyuan Zhang[1], Ge Yang[1], Guozhen Zhang[2] & Hai-Long Jiang[1]

[1] Department of Chemistry, University of Science and Technology of China, Hefei, Anhui 230026, P. R. China

[2] Department of Chemical Physics, University of Science and Technology of China, Hefei, Anhui 230026, P. R. China

[*] These authors contributed equally to this work.

Correspondence and requests for materials should be addressed to H.-L.J. (jianglab@ustc.edu.cn).

# 1. Supplementary Methods

## 1.1 Materials and Characterizations

All chemicals were purchased from commercial suppliers without further purification unless otherwise mentioned. Powder X-ray diffraction patterns (XRD) were collected on a Japan Rigaku Miniflex 600 rotation anode X-ray diffractometer equipped with graphite monochromatized Cu Kα radiation (λ = 1.54 Å). The automatic volumetric adsorption equipment (Micromeritics ASAP 2020) was performed for the $N_2$ sorption isotherms. UV-vis diffuse reflectance spectra were recorded on a UV-vis spectrophotometer (Shimadzu UV-2700). FT-IR spectra were collected on Bruker Tensor 27 IR spectrometers. The LS-55 fluorescence spectrometer made by PerkinElmer was adopted to collect the steady-state photoluminescence emission spectra. The rates of $H_2$ production were obtained by gas chromatography (Shimadzu GC-2014). The 300 W Xenon lamp (LX300F, Japan) otherwise stated was adopted as the light source. Transmission electron microscopy (TEM) images were obtained on a JEOL JEM-2100F field-emission transmission electron microscope. The Pt deposited contents were quantified on an Optima 7300 DV inductively coupled plasma atomic emission spectrometer (ICP-AES). Bruker AVANCE III 400WB spectrometer was used to obtain $^{13}C$ CP-MAS NMR spectra. Time-resolved photoluminescence decay profiles were obtained in Horiba Fluoro max plus. FLS1000 Photoluminescence Spectrometer was taken for temperature-dependent photoluminescence spectra.

## 1.2 Monomer preparation

**Synthesis of 2,5-dimethoxyterephthalaldehyde (Dma).** The synthesis was synthesized according to the previous report.[1] Typically, 1,4-dimethoxybenzene (10 g) was dissolved in dioxane (30 mL) and formaldehyde aqueous solution (5 mL) and then paraformaldehyde (3 g) was added. The mixture was stirred at 90 °C and concentrated HCl (5 mL) was added. After 30 minutes, another 5 mL of concentrated HCl was added and the solution was continued to be heated for 1 h. Then concentrated HCl (30 mL) was added and the mixture was cooled down to give a white precipitate. The solid was filtered, washed with water, dried and recrystallized with acetone to obtain 1,4-bis(chloromethyl)-2,5-dimethoxybenzene.

The obtained 1,4-bis(chloromethyl)-2,5-dimethoxybenzene (3 g) and hexamethylenetetramine (3.6 g) in 20 mL of chloroform were stirred and refluxed for 24 h. After cooling down to room temperature, the solution was filtered to afford a yellow solid. Then the solid was dried and dissolved in 4.5 mol/L aqueous solution of acetic acid (24 mL). The solution was stirred at 90 °C for 24 h, cooled down to room temperature and extracted with dichloromethane (40 mL). The organic phase is washed with water, rotary evaporated and recrystallized with ethanol to obtain bright yellow product 2,5-dimethoxyterephthalaldehyde.

**Synthesis of 2,5-dichloroterephthalaldehyde.** The synthesis was according to the previous literature.[2] Typically, 2,5-dichloro-*p*-xylene (2.5 g) was dissolved in acetic acid (21.6 mL), acetic anhydride (42 mL) and concentrated sulfuric acid (3.7 mL) while stirring and keeping the temperature at 5 °C. Chromium trioxide (4.6 g) was added

slowly to the mixture with the temperature between 5 to 12 °C (approximately 1.5 h). Then the mixture is stirred for another 5 h (10~15 °C), and afterwards, the ice water was poured into the solution and stirred thoroughly. The product was filtered out and recrystallized with ethanol to obtain 2,5-dichloroterephthalaldehyde tetra-acetate (white crystalline solid). The 2,5-dichloroterephthalaldehyde tetraacetate (0.73 g) was added into a mixture of ethanol (17 mL), water (17 mL), and concentrated sulfuric acid (1 mL) and the solution was heated to reflux for 3 h, and finally cooled down. The product was finally collected by filtration to obtain 2,5-dichloroterephthalaldehyde.

**Synthesis of 1,3,5-Tris (4-aminophenyl) benzene (TAPB).** The TAPB was synthesized according to the previous report.[3] Typically, 4-nitroacetophenone (8.3 g) was dissolved in toluene (33.3 mL) and trifluoromethane sulfonic acid (0.33 mL) was added. The mixture was stirred at 125 °C and refluxed for 48 h, and then the mixture was cooled down to give a black precipitate. The solid was filtered, and washed with DMF by refluxing for two times. Following that, the solid was filtered and dried to obtain 1,3,5-tris(4-nitrophenyl)benzene.

The obtained 1,3,5-tris(4-nitrophenyl)benzene (4 g) and Pd/C (5 wt%, 1.6 g) were dispersed in ethanol (80 mL). After the mixture was heated to 90 °C for 5 min, hydrazine hydrate (12 mL) was added, and the solution was refluxed overnight. The solution was then filtered through celite at the high temperature, before cooling down to crystallize the product. The solid was finally filtered, and washed with a little ethanol to obtain 1,3,5-tris(4-aminophenyl)benzene.

**Synthesis of 1,3,5-Tris (4-aminophenyl) triazine (TAPT).** The TAPT was synthesized

according to the previous report.[4] Typically, 4-aminobenzonitrile (0.77 g) was cooled in ice water bath, and trifluoromethane sulfonic acid (2 mL) was added and stirred at room temperature under Ar atmosphere. After 24 h, 20 mL water was added, and the mixture was neutralized to pH at 9~10 with 2 mol/L aqueous NaOH. The solid was finally filtered, and washed with water to afford bright yellow product 1,3,5-tris(4-aminophenyl)triazine.

## 1.3 Photoelectrochemical Measurements

The CHI 760E electrochemical workstation from Chenhua Instrument was employed in a standard three-electrode system with the photocatalyst-coated FTO as a working electrode (size: 20*40*2.2 mm; coated area: 20*20 mm), Pt plate as a counter electrode, and an Ag/AgCl electrode as a reference electrode for photocurrent measurement. The 300 W Xenon lamp with a UV cut-off filter (>380 nm) was adopted as the light source. The 2 mg catalysts in 2 mL methanol were mixed with 10 μL Nafion. The mixture was dispersed uniformly by ultrasonic and dropped on FTO. A 0.1 M $Na_2SO_4$ solution was used as the electrolyte. The photoreactive signals were recorded under chopped light with at + 0.5 V. The switch of the light is realized by a cardboard covered with tinfoil. EIS and Mott-Schottky plot measurements were carried out in a conventional three electrode cell with a Zahner Zennium electrochemical workstation, where Ag/AgCl electrode as the reference electrode, the glassy carbon as the working electrode (area: 0.2 $cm^2$) and Pt plate as the counter electrode were used. The electrolyte was the same as that in the photocurrent measurement. The 2 mg catalysts in 2 ml methanol were mixed with 10 μL Nafion. Then, 30 μL suspension was dropped on the glassy carbon electrode. EIS was performed at -1.5 V in a frequency range from $10^{-1}$ to $10^5$ Hz.

### 1.4 Supplementary Calculation Details

Density functional theory (DFT) using gaussian 16 software was used for the calculation of the exciton binding energy ($E_b$) by subtracting the optical gap ($E_{opt}$) from the fundamental energy gap ($E_{fund}$).[5,6] The $E_{opt}$ corresponding to the energy of the lowest electronic transition accessible via absorption of a single photon, was obtained from the time-dependent DFT (TD-DFT) calculations. The $E_{fund}$ is defined as the energy difference between the ionization potential IP (IP = $E_{total}$ (cation) − $E_{total}$ (neutral)) and the electron affinity EA (EA = $E_{total}$ (neutral) – $E_{total}$ (anion)).[7] The $E_b$, $E_{opt}$, IP, and EA obtained using M06-2X/6-31G(d,p) are derived in the geometry optimization of the neutral molecule with ωB97XD/6-31G(d,p). The charge density difference map was constructed by the Multiwfn program and Visual Molecular Dynamics (VMD).[8,9]

The original charge density between the ground states (GS) and the excited states (ES) was generated using the following formula:

$$\Delta\rho(r)=\rho_{ES}(r)-\rho_{GS}(r)$$

To visualize the charge transfer more clearly, another intuitive representation of charge density difference has been presented.[10] Two centroids of charges ($C_+$ and $C_-$) associated with the positive and negative density regions are defined. Firstly, the root-mean-square deviations (rmsd) along the three axes ( $\sigma_{aj}$, $j = x,y,z$; $a = +$ or - ) are computed as

$$\sigma_{aj}=\sqrt{\frac{\sum_i \rho_a(r_i)(j_i-j_a)^2}{\sum_i \rho_a(r_i)}}$$

Then the two centroids can then simply be defined as

$$C_+(r)=A_+e\left(-\frac{(x-x_+)^2}{2\sigma_{+x}^2}-\frac{(y-y_+)^2}{2\sigma_{+y}^2}-\frac{(z-z_+)^2}{2\sigma_{+z}^2}\right)$$

$$C_-(r)=A_-e\left(-\frac{(x-x_-)^2}{2\sigma_{-\mathrm{x}}^2}-\frac{(y-y_-)^2}{2\sigma_{-\mathrm{y}}^2}-\frac{(z-z_-)^2}{2\sigma_{-\mathrm{z}}^2}\right)$$

These are normalized three dimensional gaussian distributions placed on the center of each density difference, scaled by the rmsd of this function.

The S index represents the overlap integral of hole-electron distribution, evaluated using the following equation:

$$S_{\mathrm{index}}=\int\sqrt{\rho^{\mathrm{hole}}(r)\rho^{\mathrm{ele}}(r)}\mathrm{dr}$$

Where $\rho^{\mathrm{hole}}$ and $\rho^{\mathrm{ele}}$ stand for the density distribution of hole and electron, respectively.

The D index reflects the distance between the centroid of hole and electron, measured using the following equation:

$$D_{\mathrm{x}}=|X_{\mathrm{ele}}-X_{\mathrm{hole}}|$$

$$D_{\mathrm{y}}=|Y_{\mathrm{ele}}-Y_{\mathrm{hole}}|$$

$$D_{\mathrm{z}}=|Z_{\mathrm{ele}}-Z_{\mathrm{hole}}|$$

$$D_{index}=\sqrt{(D_{\mathrm{x}})^2+(D_{\mathrm{y}})^2+(D_{\mathrm{z}})^2}$$

Where $X_{\mathrm{hole}}$ corresponds to the $X$ coordinate of the centroid of electron obtained through multiplying the $\rho^{\mathrm{hole}}$ function by the $X$ coordinate variable and integrating in the whole space.

The $D_{\mathrm{CT}}$ characterizes the total CT length between the negative and positive barycenters, estimated using the following equation:

$$D_{\mathrm{x}}=|X_+-X_-|$$

$$D_y = |Y_+ - Y_-|$$

$$D_z = |Z_+ - Z_-|$$

$$D_{CT} = \sqrt{(D_x)^2 + (D_y)^2 + (D_z)^2}$$

The $D_{index}$ is evaluated as the distance between the centroid of electron and hole distributions, it is not the same as the $D_{CT}$ calculated as centroid distance between positive and negative parts of density difference between relaxed excited state density and ground state density.

## 2. Supplementary Figures 1-42

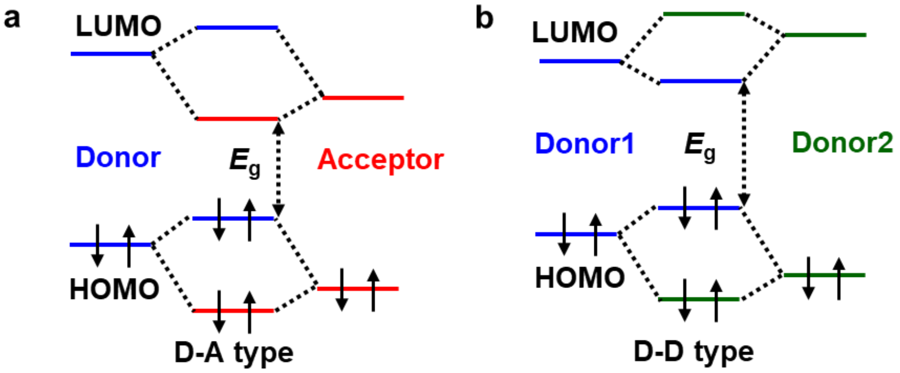


**Supplementary Figure 1 | Two types of bandgaps ($E_g$) in COFs and the corresponding energy level alignment of frontier orbitals in building blocks:** (a) D-A type; (b) D-D type.

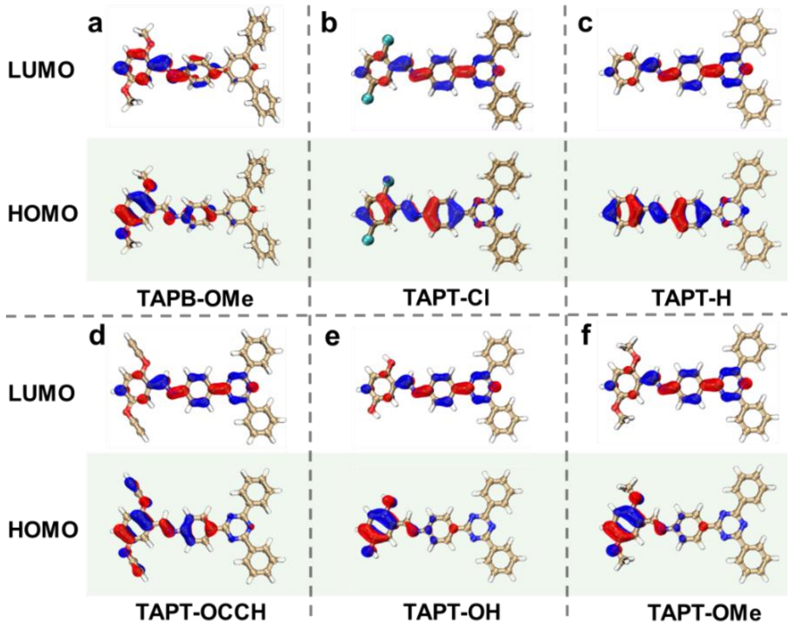


**Supplementary Figure 2 | Visualization of the HOMO (bottom) and LUMO (top) for the D-A pairs of** (a) TAPB-OMe, (b) TAPT-Cl, (c) TAPT-H, (d) TAPT-OCCH, (e) TAPT-OH and (f) TAPT-OMe (Isovalue = 0.04 a.u.).

From the distribution of HOMO and LUMO in D-A pairs, the HOMO and LUMO are mainly located in the aldehyde- (the left part of the pair) and amine- (the right part of the pair) based segments, respectively, for TAPT-H, TAPT-OCCH, TAPT-OH and TAPT-OMe, indicating the effective separation of HOMO and LUMO. In contrast, TAPB-OMe and TAPT-Cl exhibit a high overlap between the HOMO and LUMO.

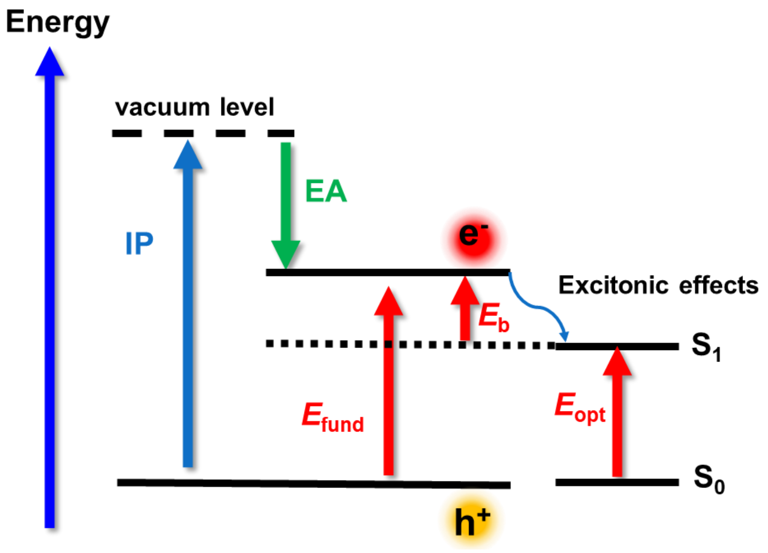


**Supplementary Figure 3 | Illustration of various energy gaps in the molecular base:** $S_0$ and $S_1$ denote the electronic ground state and the lowest excited state, respectively. IP: ionization potential; EA: electron affinity; $E_{fund}$: fundamental gap; $E_{opt}$: optical gap; $E_b$: exciton binding energy.

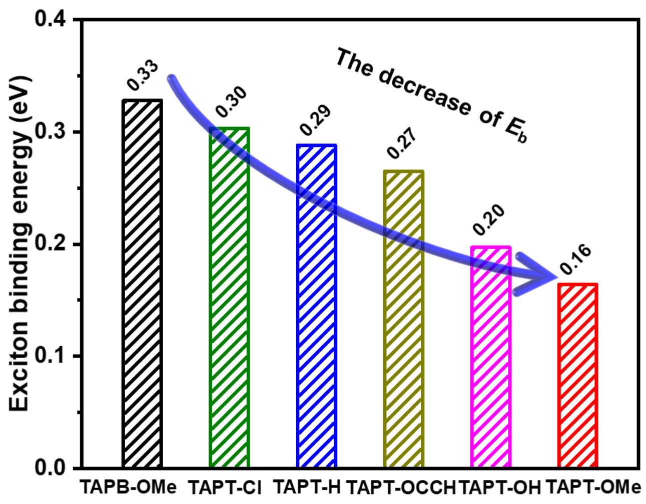


**Supplementary Figure 4 | Trend of calculated exciton binding energy for the D-A pairs.**

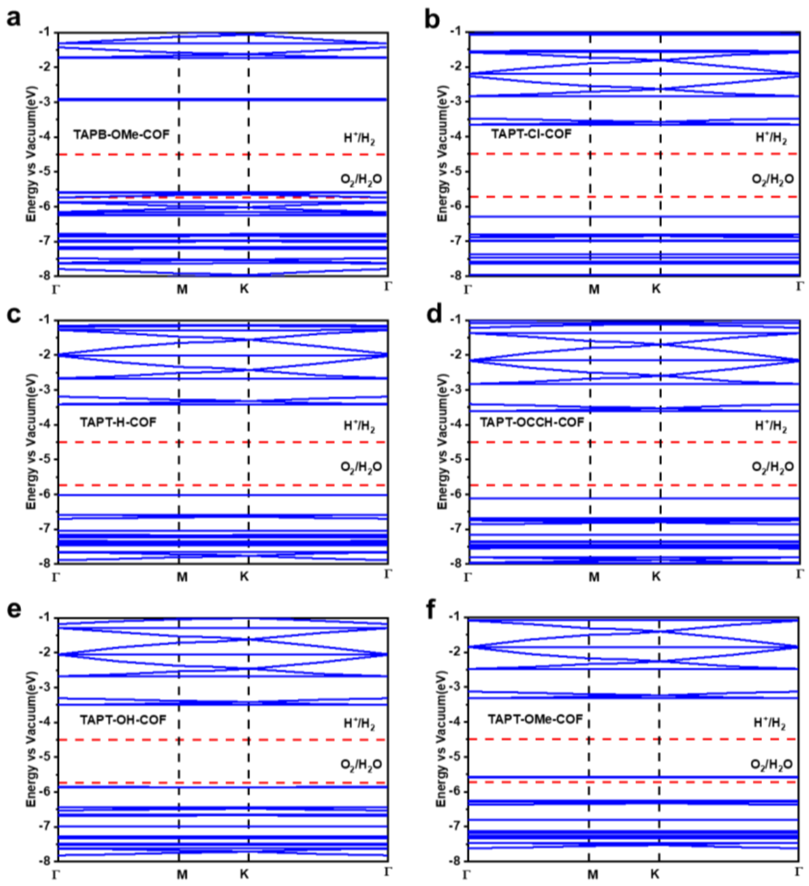


**Supplementary Figure 5 | Calculated electronic band structures relative to vacuum levels with HSE06 method of** (a) TAPB-OMe-COF, (b) TAPT-Cl-COF, (c) TAPT-H-COF, (d) TAPT-OCCH-COF, (e) TAPT-OH-COF and (f) TAPT-OMe-COF. The red dashed lines denote redox potential of water at pH = 0.

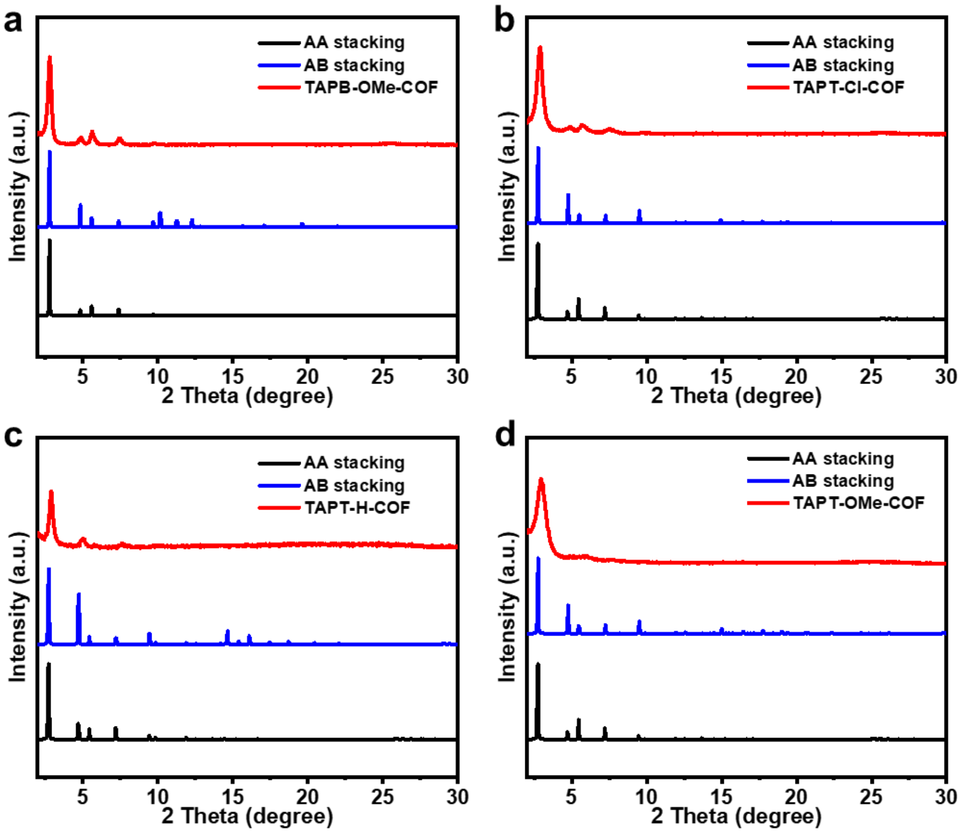


**Supplementary Figure 6 | Powder XRD profiles of (a) TAPB-OMe-COF, (b) TAPT-Cl-COF, (c) TAPT-H-COF, (d) TAPT-OMe-COF** were presentited through experimental observation (in red), as well as simulation using the AA stacking mode (in black) and the staggered AB stacking mode (in blue).

For the corresponding D-A COFs, the simulated Powder XRD pattern of the AA stacking mode (black curve) matches the observed peak positions and intensities, while the staggered AB stacking mode (blue curve) fails to reproduce the experimental pattern (red curve).

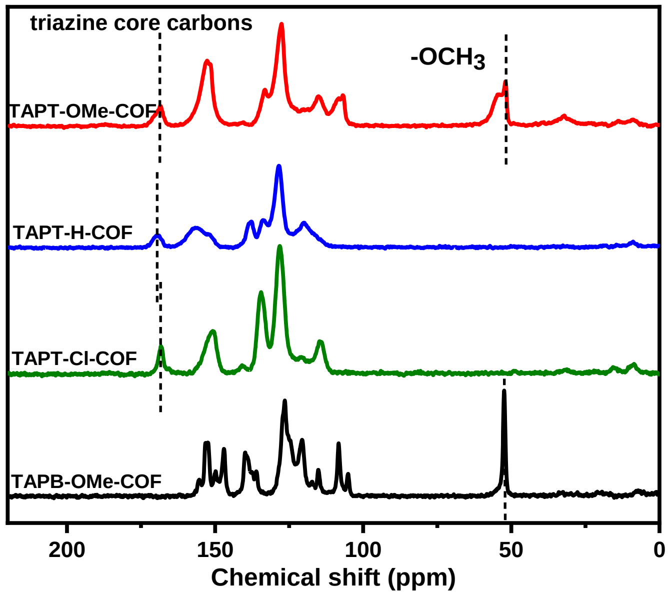


**Supplementary Figure 7 | $^{13}C$ CP-MAS NMR spectra of D-A COFs.**

According to the $^{13}C$-NMR results, the carbon resonance at ~52 ppm is associated with the methoxyl carbon of TAPB-OMe-COF and TAPT-OMe-COF. The peak at 168-170 ppm can be attributed to the triazine core carbons, which can be clearly found in TAPT-OMe-COF, TAPT-H-COF and TAPT-Cl-COF. The other peaks at 151-156 and 105-152 ppm can be assigned to the -C=N and aromatic carbons, respectively.[11]

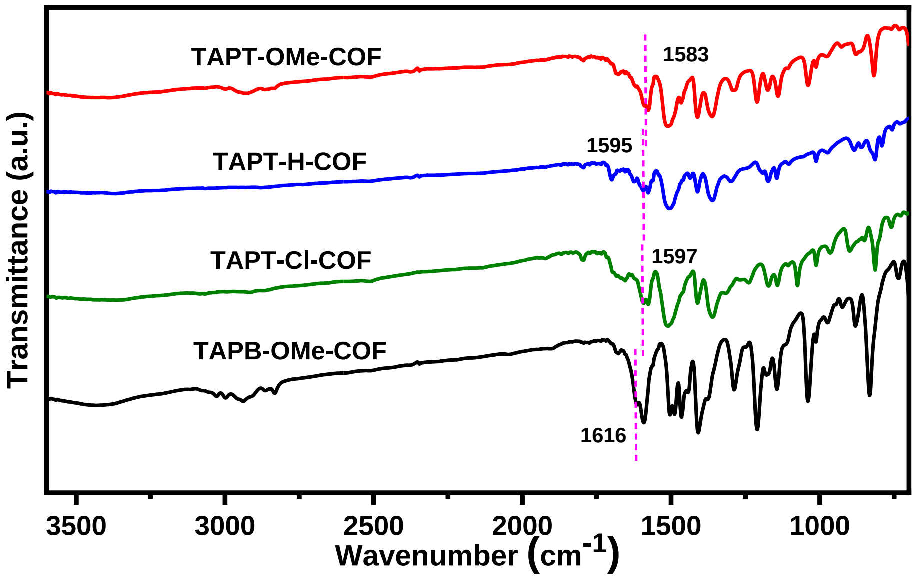


**Supplementary Figure 8 | FT-IR spectra of TAPT-OMe-COF, TAPT-H-COF, TAPT-Cl-COF and TAPB-OMe-COF.**

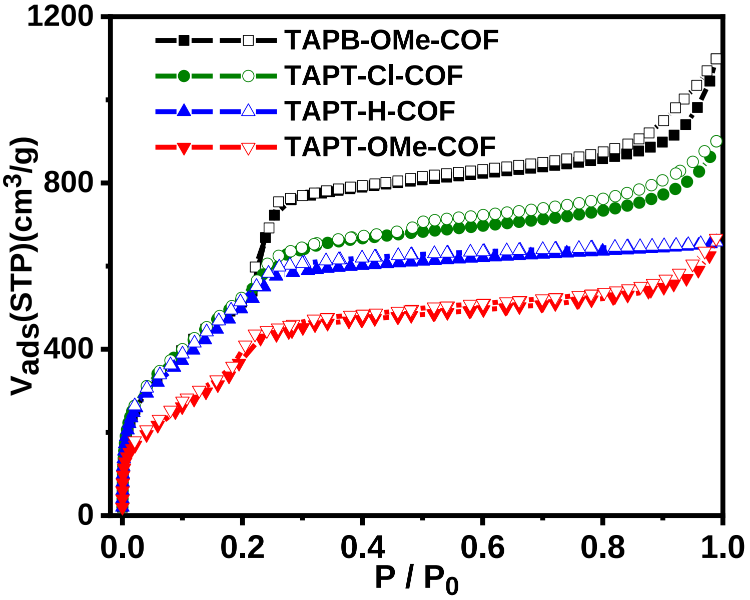


**Supplementary Figure 9 | $N_2$ sorption isotherms for different D-A COFs at 77 K.** Filled and open symbols represent adsorption and desorption branches, respectively. The BET surface areas of TAPB-OMe-COF, TAPT-Cl-COF, TAPT-H-COF and TAPT-OMe-COF are determined to be 2708, 2147, 1987 and 1618 $m^2/g$, respectively.

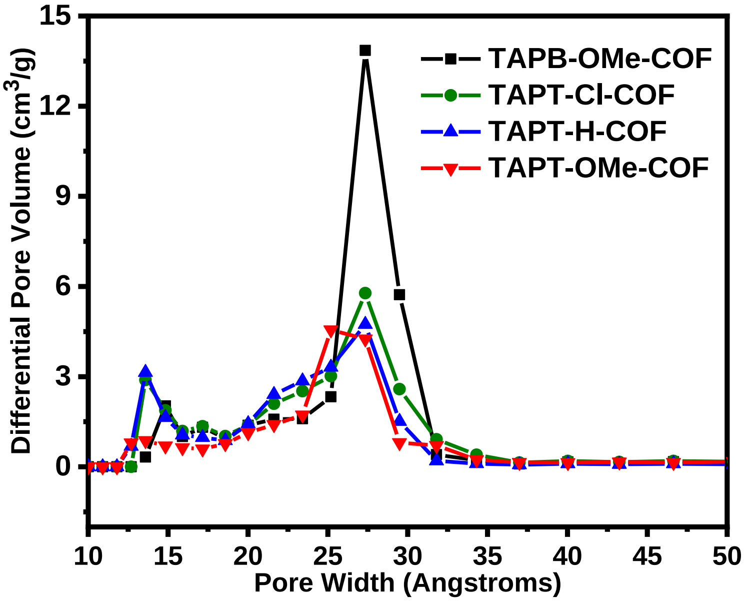


**Supplementary Figure 10 | Pore size distribution of different D-A COFs** based on the above $N_2$ sorption data (**Supplementary Fig. 9**) and the DFT model, suggesting that the main pore sizes for TAPB-OMe-COF, TAPT-Cl-COF, TAPT-H-COF and TAPT-OMe-COF are around 2.7, 2.7, 2.7 and 2.5 nm, respectively.

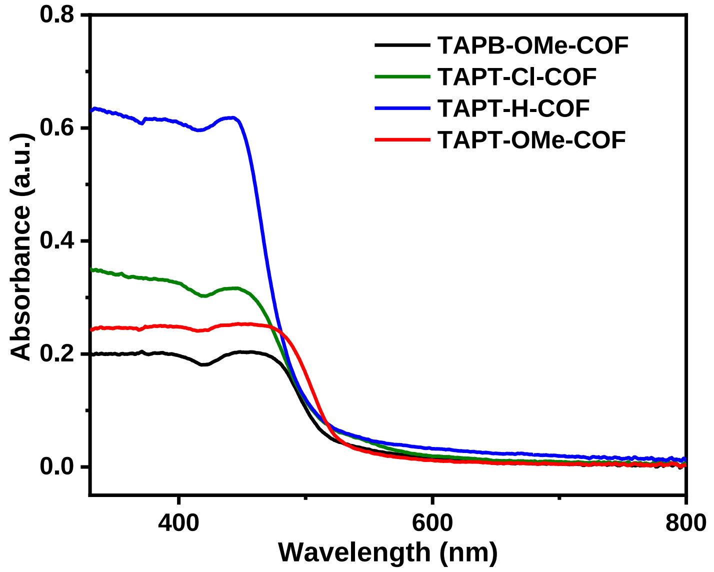


**Supplementary Figure 11 | UV-vis spectra of the D-A COFs.**

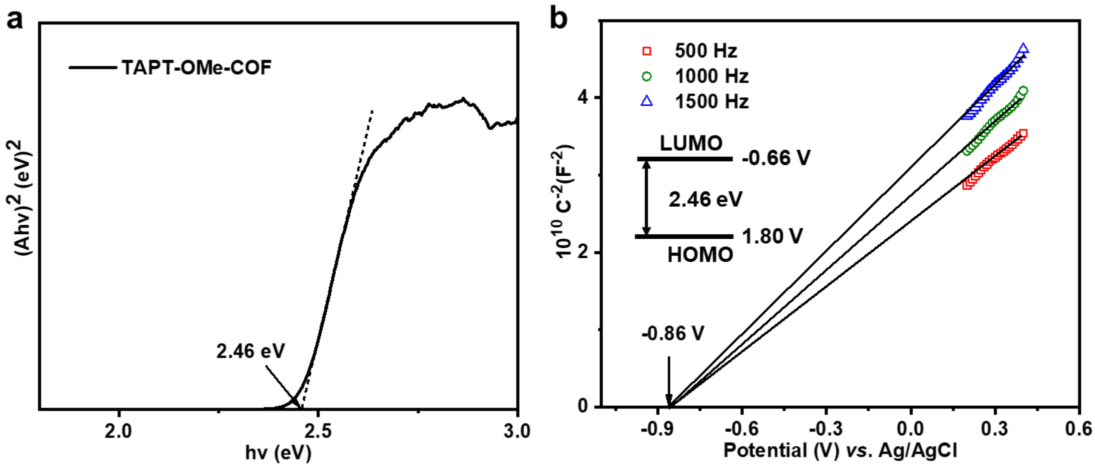


**Supplementary Figure 12 |** (a) Tauc plot of TAPT-OMe-COF. (b) Mott-Schottky plots for TAPT-OMe-COF in 0.1 M $Na_2SO_4$ aqueous solution. Inset: the energy diagram of TAPT-OMe-COF.

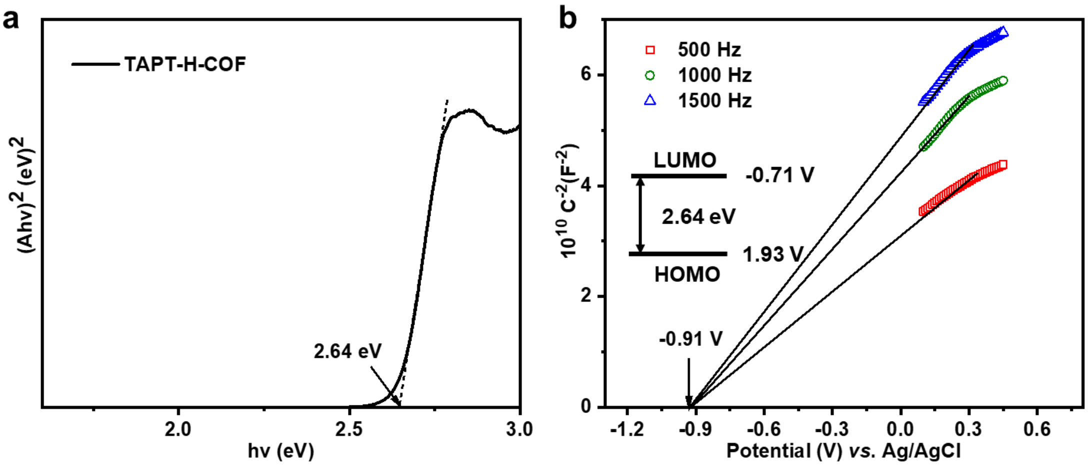


**Supplementary Figure 13 |** (a) Tauc plot of TAPT-H-COF. (b) Mott-Schottky plots for TAPT-H-COF in 0.1 M $Na_2SO_4$ aqueous solution. Inset: the energy diagram of TAPT-H-COF.

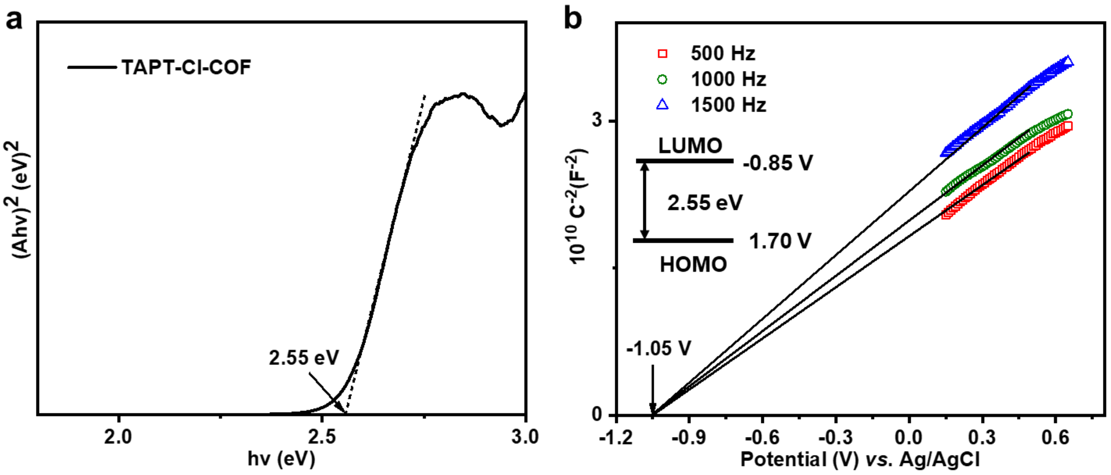


**Supplementary Figure 14 |** (a) Tauc plot of TAPT-Cl-COF. (b) Mott-Schottky plots for TAPT-Cl-COF in 0.1 M $Na_2SO_4$ aqueous solution. Inset: the energy diagram of TAPT-Cl-COF.

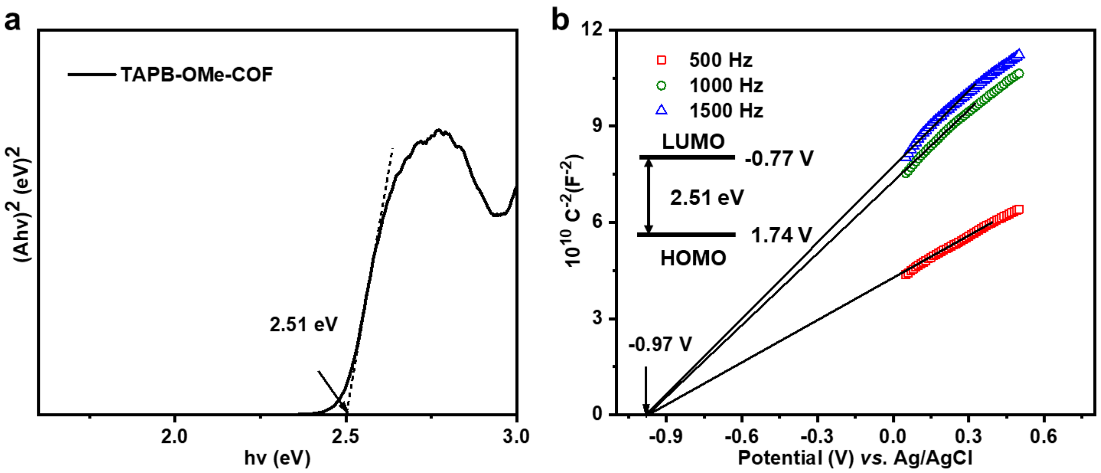


**Supplementary Figure 15 |** (a) Tauc plot of TAPB-OMe-COF. (b) Mott-Schottky plots for TAPB-OMe-COF in 0.1 M $Na_2SO_4$ aqueous solution. Inset: the energy diagram of TAPB-OMe-COF.

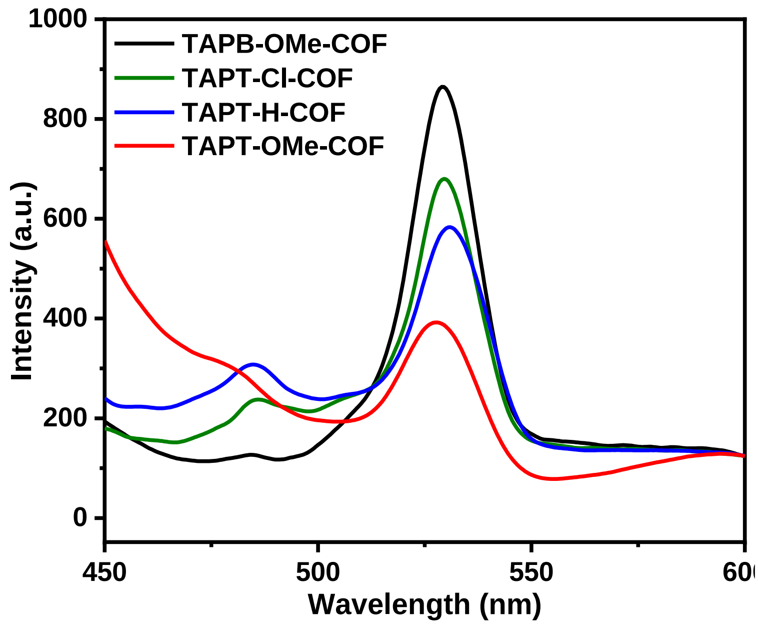


**Supplementary Figure 16 | Fluorescence emission spectra for the D-A COFs under excitation at 380 nm.**

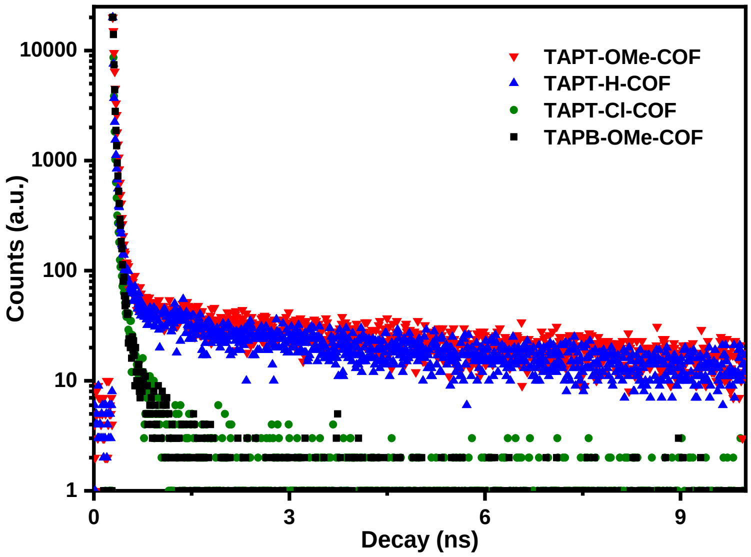


**Supplementary Figure 17 | Time-resolved PL decay profiles for D-A COFs (excitation at 367 nm, emission at 530 nm).**

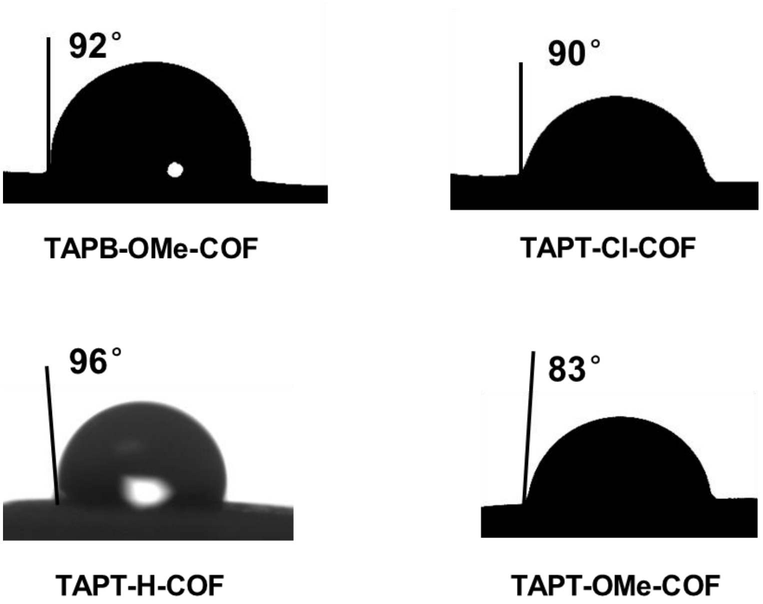


**Supplementary Figure 18 | Static water contact angles of D-A COFs.**

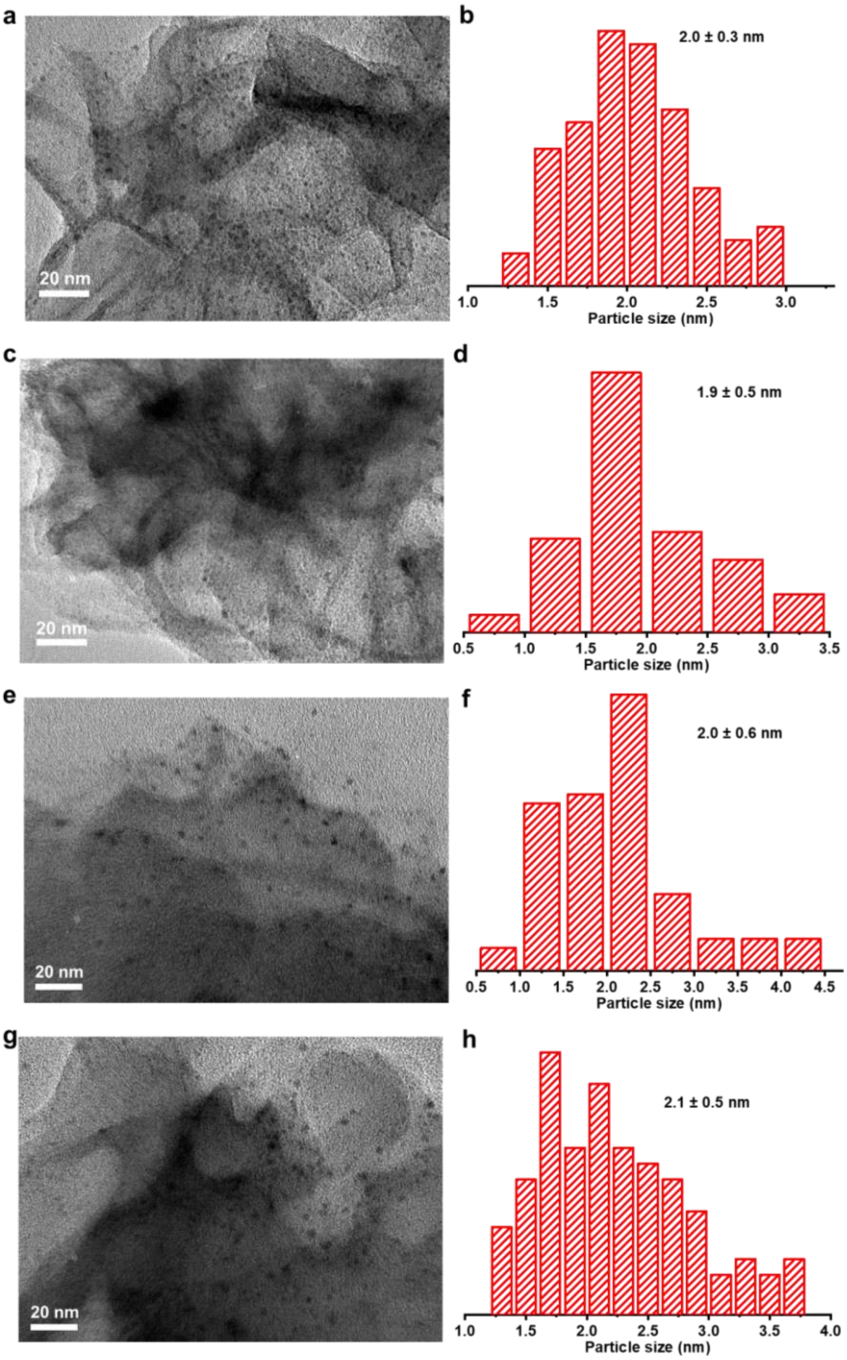


**Supplementary Figure 19 | (a, c, e, g) TEM images and (b, d, f, h) the corresponding Pt size distributions of (a, b) TAPB-OMe-COF, (c, d) TAPT-Cl-COF, (e,f) TAPT-H-COF, and (g, h) TAPT-OMe-COF after the reaction.**

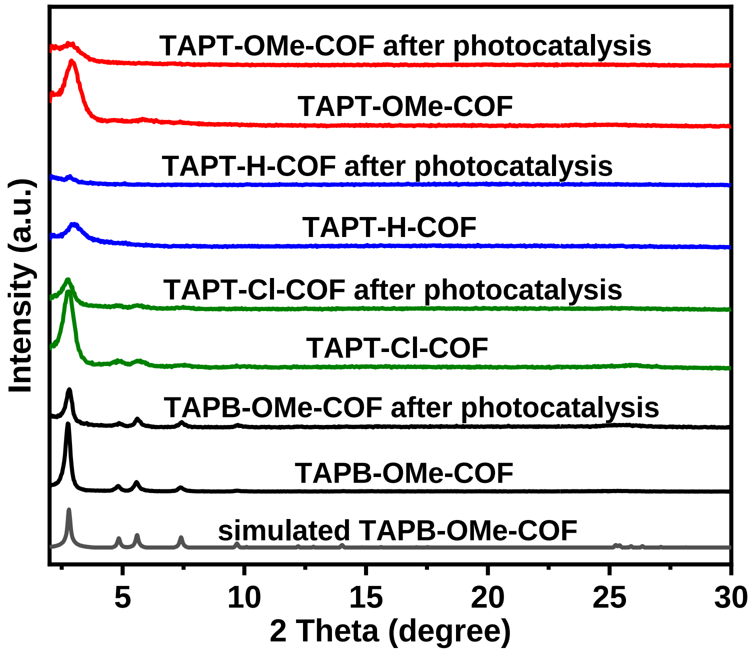


**Supplementary Figure 20 | Powder XRD patterns for the D-A COFs after photocatalytic $H_2$ production tests.**

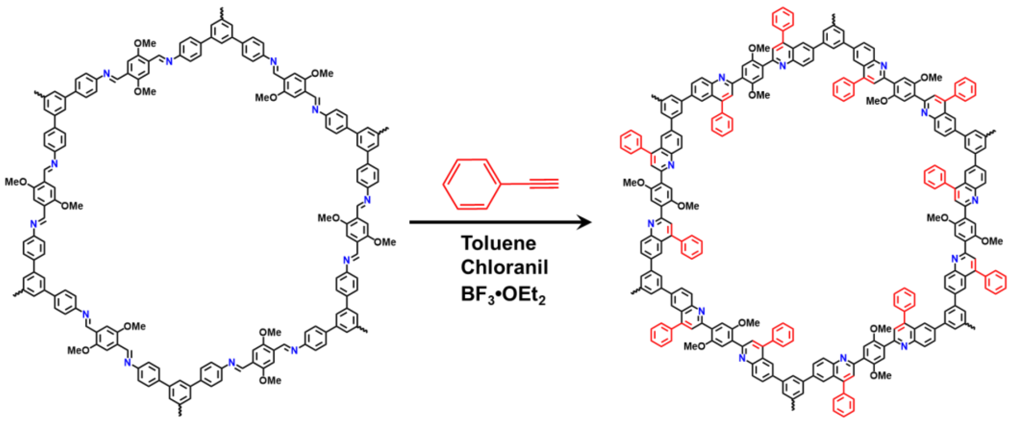


**Supplementary Figure 21 | The reaction scheme for post-synthetic modification of COFs via aza-Diels-Alder reaction (TAPB-OMe-COF as a representative).**

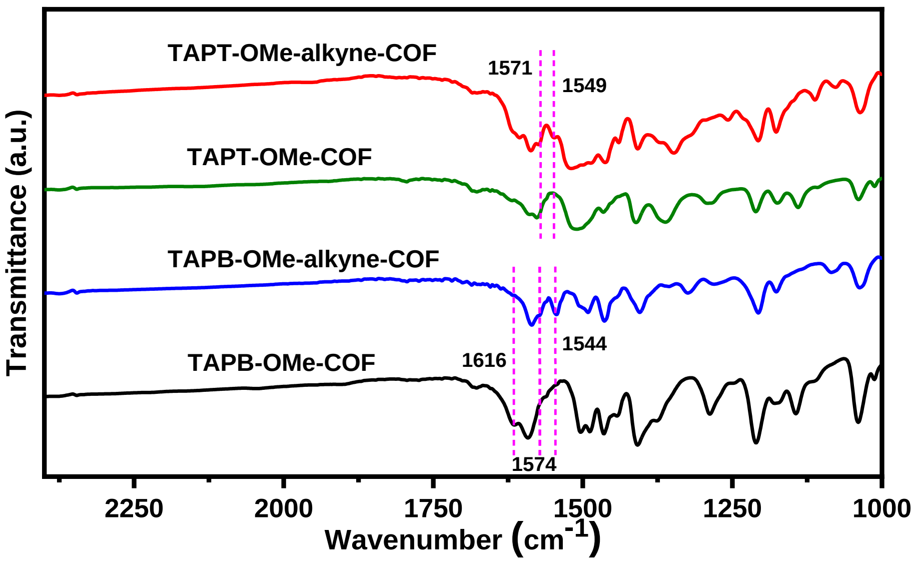


**Supplementary Figure 22 | FT-IR spectra of TAPT-OMe-COF and TAPB-OMe-COF before and after the post-synthetic modification with phenylacetylene.**

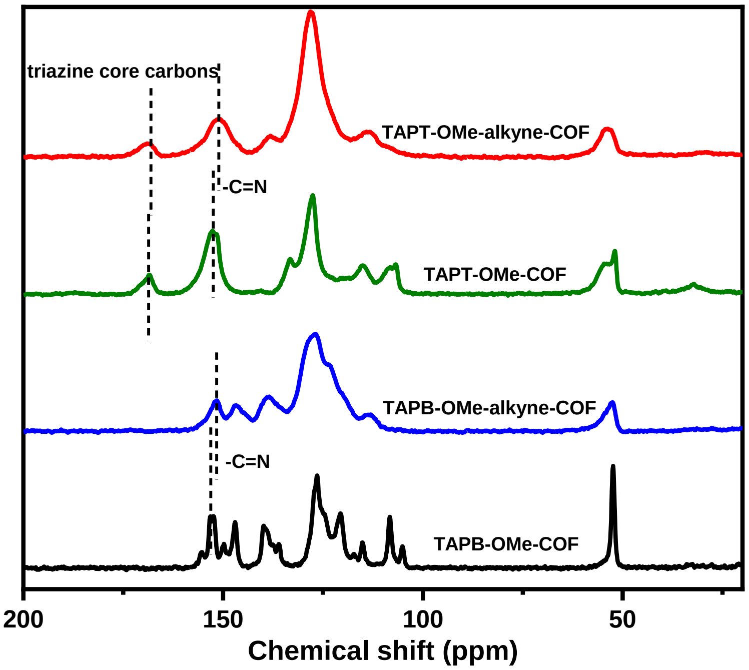


**Supplementary Figure 23 | $^{13}C$ CP-MAS NMR spectra of TAPB-OMe-COF and TAPT-OMe-COF before and after post-synthetic modification with phenylacetylene.**

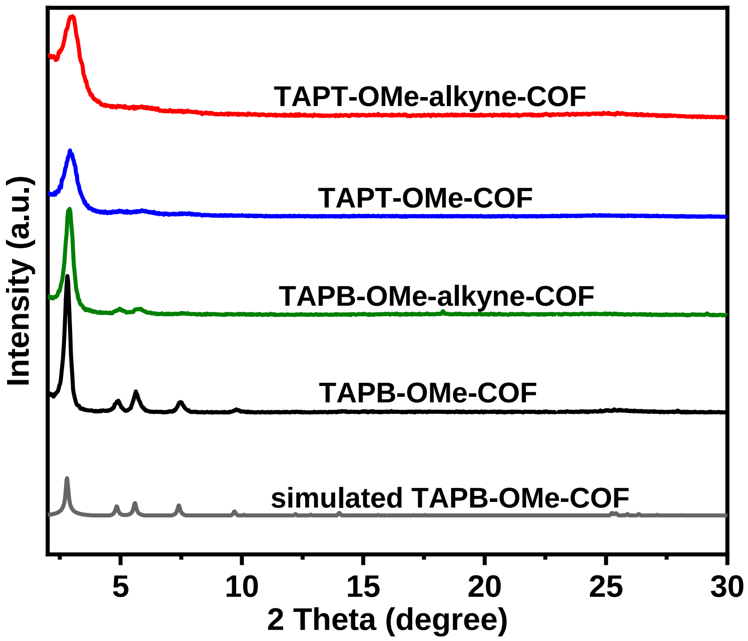


**Supplementary Figure 24 | Powder XRD patterns for TAPT-OMe-COF and TAPB-OMe-COF before and after the post-synthetic modification with phenylacetylene.**

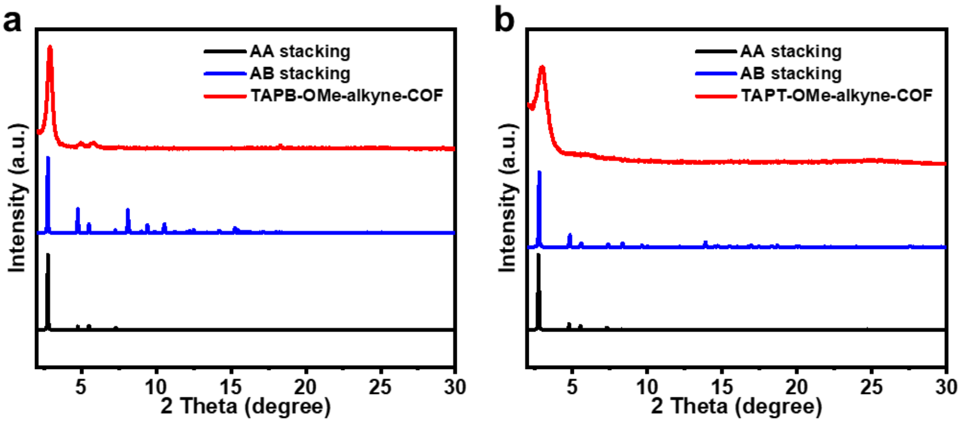


**Supplementary Figure 25 | Powder XRD profiles of (a) TAPB-OMe-alkyne-COF and (b) TAPT-OMe-alkyne-COF** were presented through experimental observation (in red), as well as simulation using the AA stacking mode (in black) and the staggered AB stacking mode (in blue).

Post-synthetic modification does not alter the pristine stacking mode of COFs, as they continue to stack in the AA stacking mode.

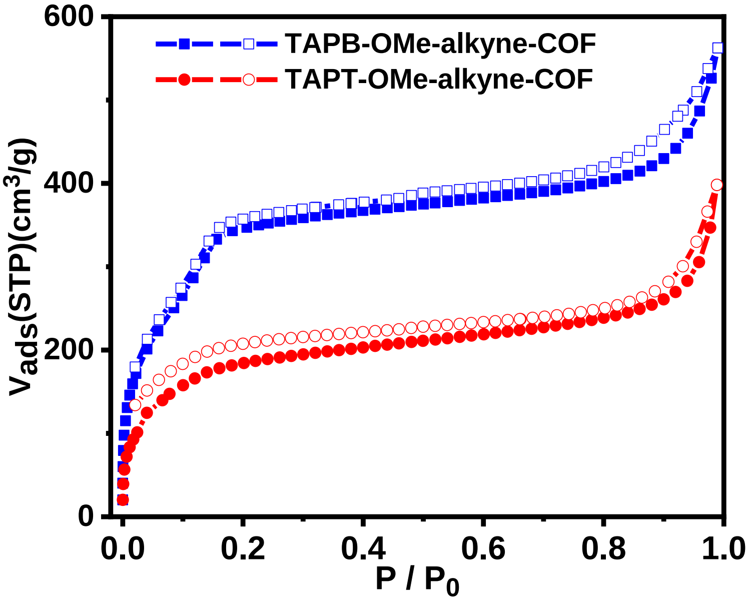


**Supplementary Figure 26 | $N_2$ sorption isotherms for TAPB-OMe-alkyne-COF and TAPT-OMe-alkyne-COF at 77 K.** Filled and open symbols represent adsorption and desorption branches, respectively. The BET surface areas of TAPB-OMe-alkyne-COF and TAPT-OMe-alkyne-COF are determined as 1169 and 609 $m^2/g$, respectively.

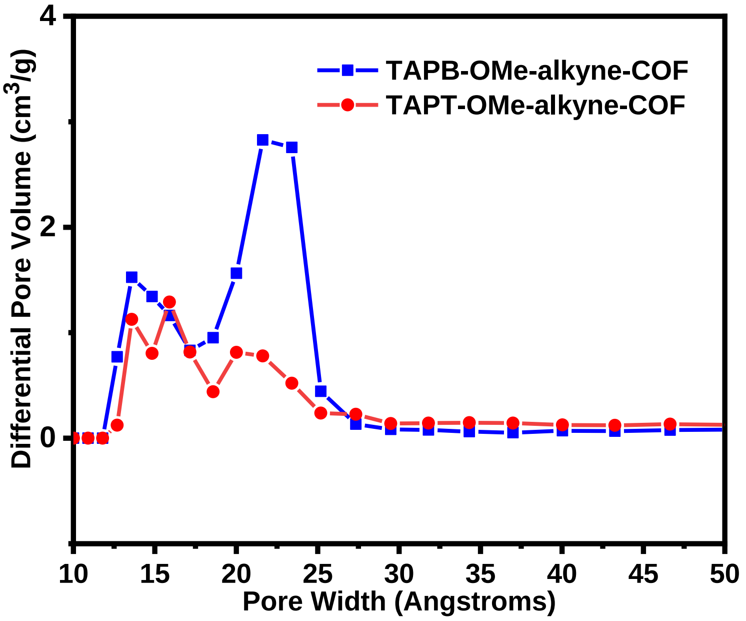


**Supplementary Figure 27 | Pore size distributions of TAPB-OMe-alkyne-COF and TAPT-OMe-alkyne-COF** based on the above $N_2$ sorption data (**Supplementary Fig. 26**) and the DFT model, indicating that the main pore sizes of TAPB-OMe-alkyne-COF and TAPT-OMe-alkyne-COF are centered at 2.1 and 1.7 nm, respectively.

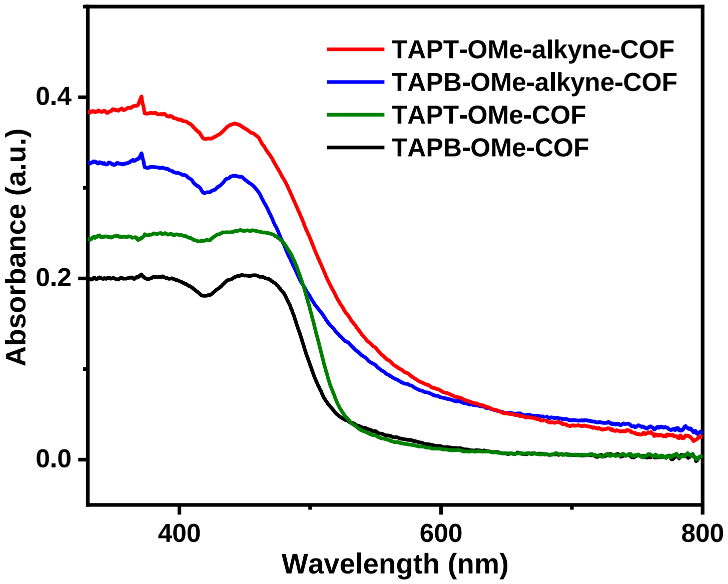


**Supplementary Figure 28 | UV-vis spectra of the TAPB-OMe-COF and TAPT-OMe-COF before and after post-synthetic modification with phenylacetylene.**

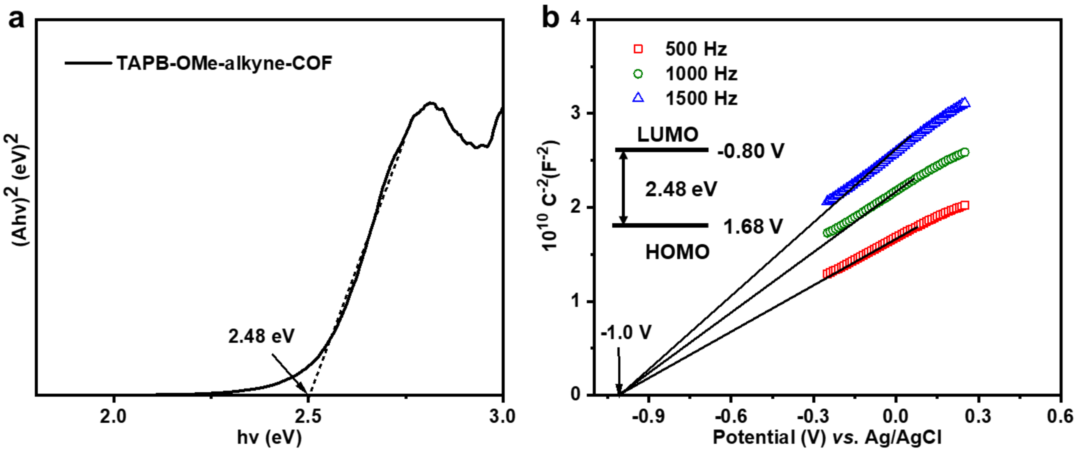


**Supplementary Figure 29 |** (a) Tauc plot of TAPB-OMe-alkyne-COF. (b) Mott-Schottky plots for TAPB-OMe-alkyne-COF in 0.1 M $Na_2SO_4$ aqueous solution. Inset: the energy diagram of TAPB-OMe-alkyne-COF.

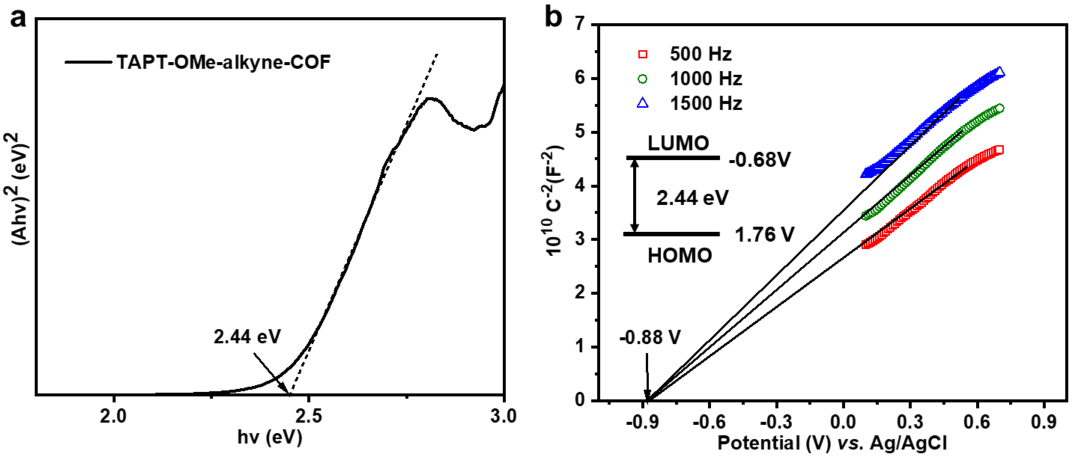


**Supplementary Figure 30 |** (a) Tauc plot of TAPT-OMe-alkyne-COF. (b) Mott-Schottky plots for TAPT-OMe-alkyne-COF in 0.1 M $Na_2SO_4$ aqueous solution. Inset: the energy diagram of TAPT-OMe-alkyne-COF.

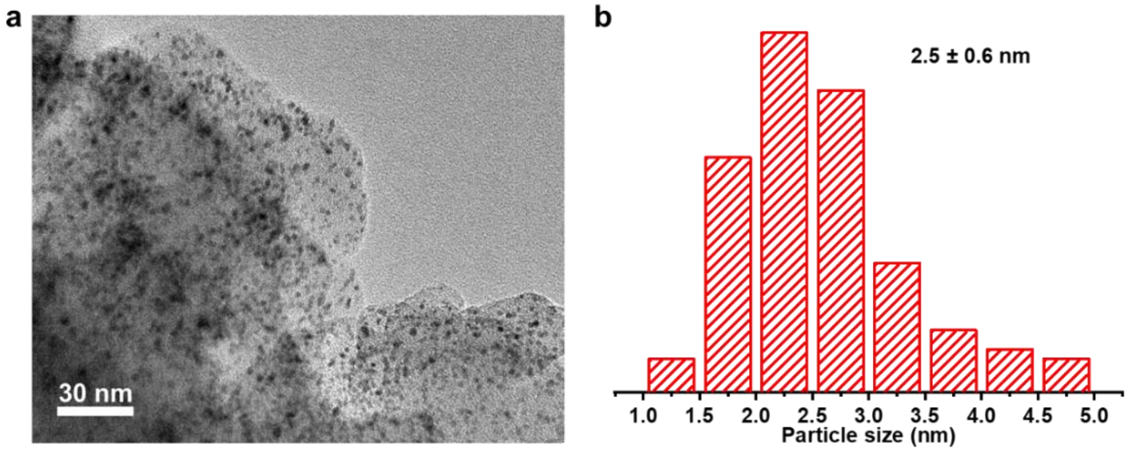


**Supplementary Figure 31 |** (a) TEM images of TAPT-OMe-alkyne-COF with *in situ* deposited Pt nanoparticles after 3 photocatalytic cycles. (b) Size distribution of the *in situ* deposited Pt nanoparticles.

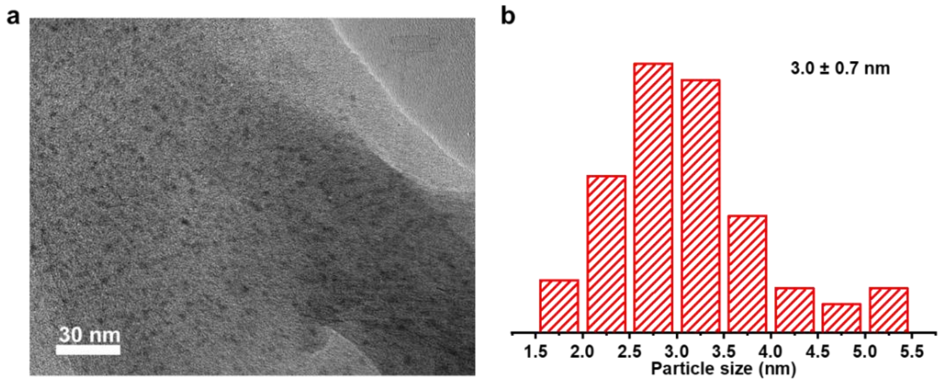


**Supplementary Figure 32 |** (a) TEM images of TAPB-OMe-alkyne-COF with *in situ* deposited Pt nanoparticles after 3 photocatalytic cycles. (b) Size distribution of the *in situ* deposited Pt nanoparticles.

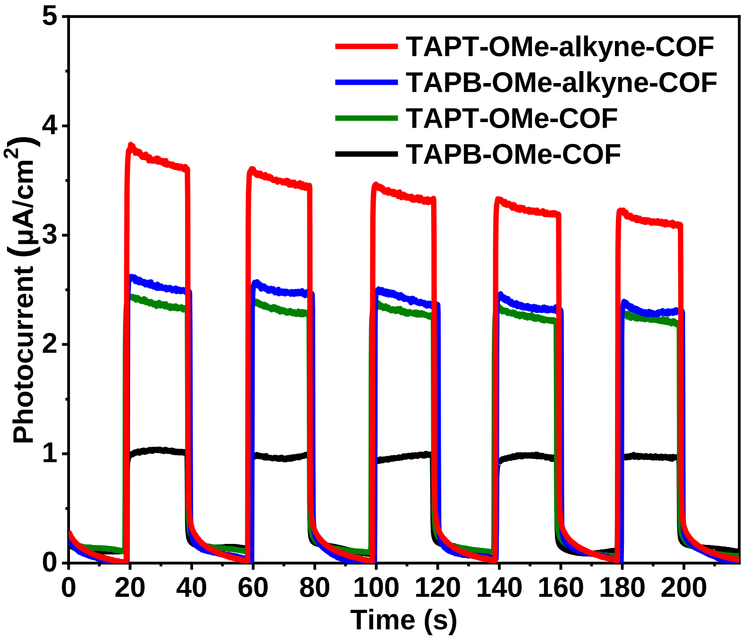


**Supplementary Figure 33 | Photocurrent responses of TAPB-OMe-COF and TAPT-OMe-COF before and after post-synthetic modification with phenylacetylene**.

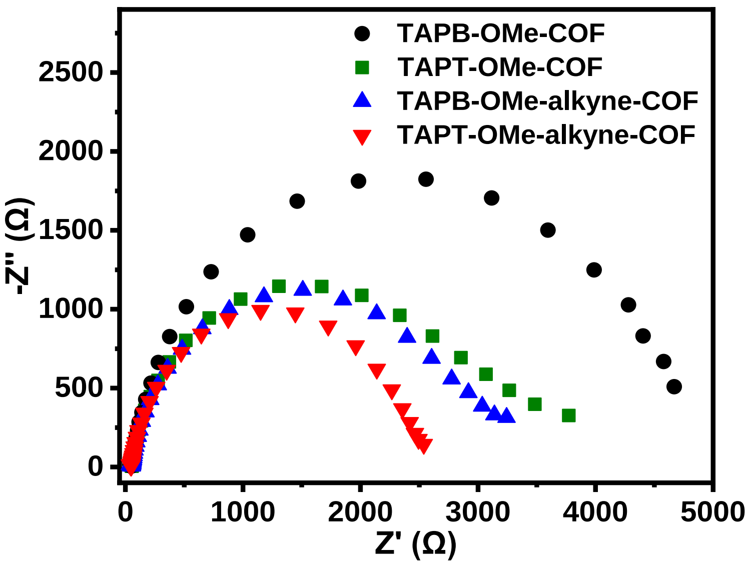


**Supplementary Figure 34 | EIS Nyquist plots of the TAPB-OMe-COF and TAPT-OMe-COF before and after post-synthetic modification with phenylacetylene.**

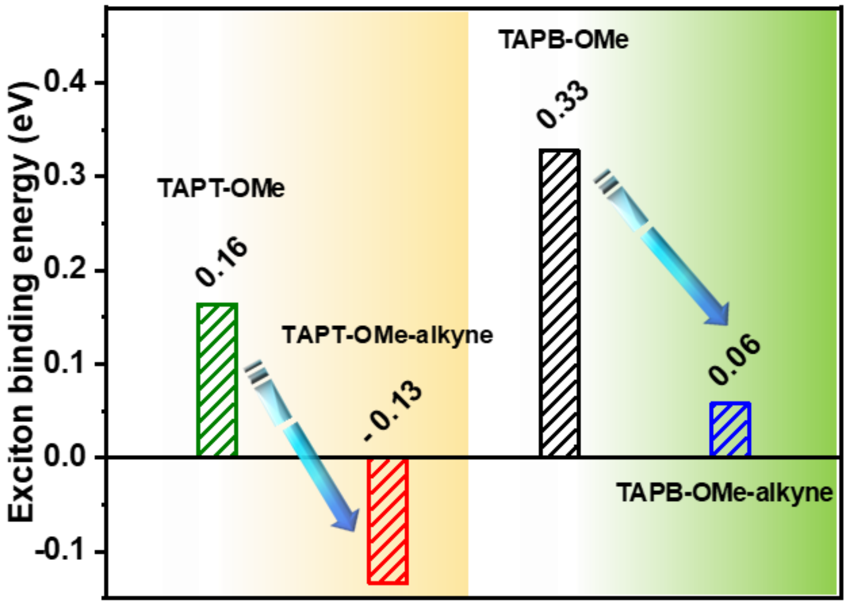


**Supplementary Figure 35 | Calculated exciton binding energy $E_b$ for TAPT-OMe and TAPB-OMe before and after post-synthetic modification with phenylacetylene.**

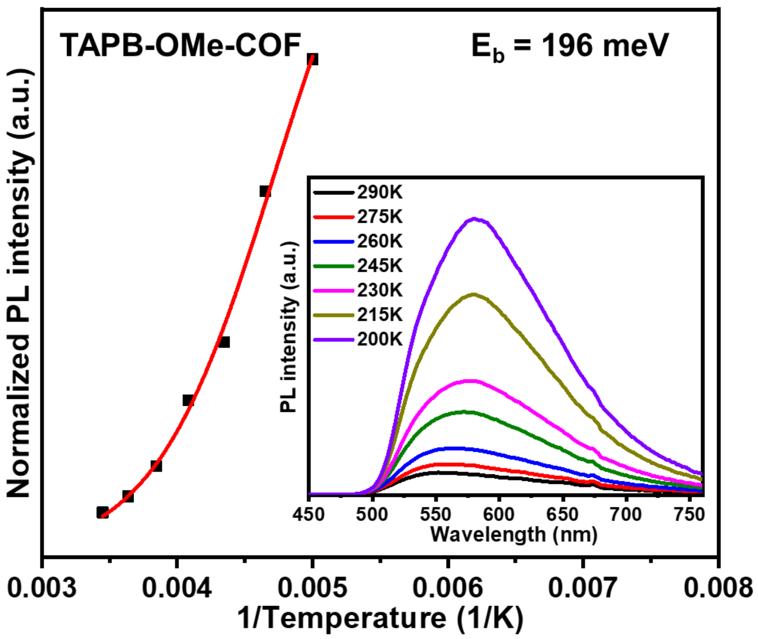


**Supplementary Figure 36 |** Integrated photoluminescence emission intensity as a function of temperature (Inset: temperature-dependent photoluminescence spectra, excitation at 400 nm) of TAPB-OMe-COF. $E_b$ is derived from the Arrhenius equation.

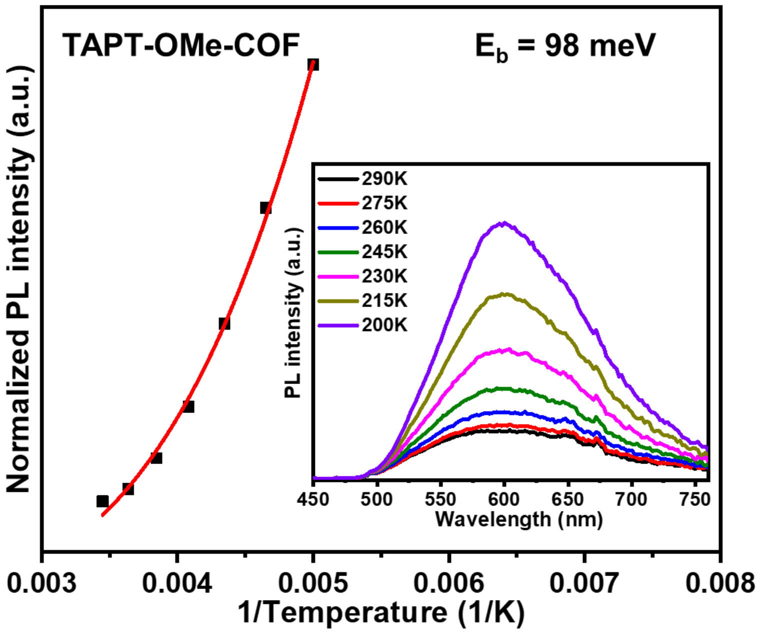


**Supplementary Figure 37 |** Integrated photoluminescence emission intensity as a function of temperature (Inset: temperature-dependent photoluminescence spectra, excitation at 400 nm) of TAPT-OMe-COF. $E_b$ is derived from the Arrhenius equation.

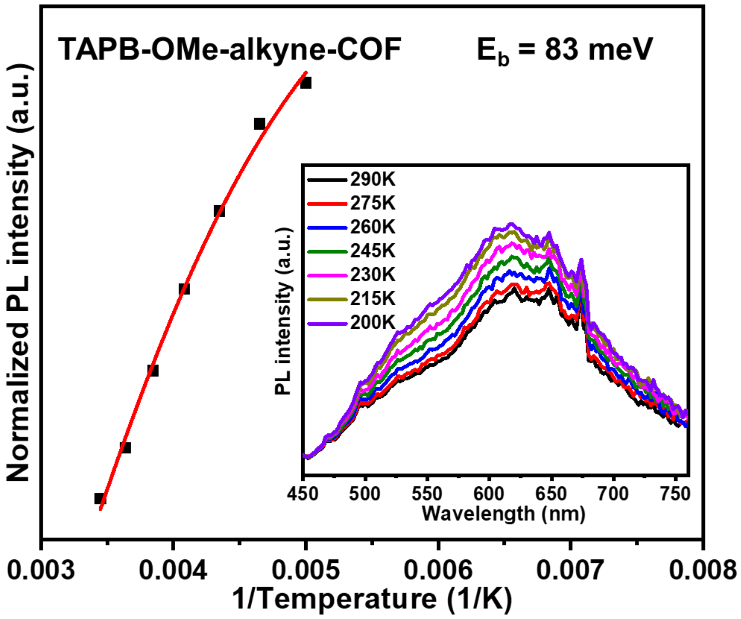


**Supplementary Figure 38 |** Integrated photoluminescence emission intensity as a function of temperature (Inset: temperature-dependent photoluminescence spectra, excitation at 400 nm) of TAPB-OMe-alkyne-COF. $E_b$ is derived from the Arrhenius equation.

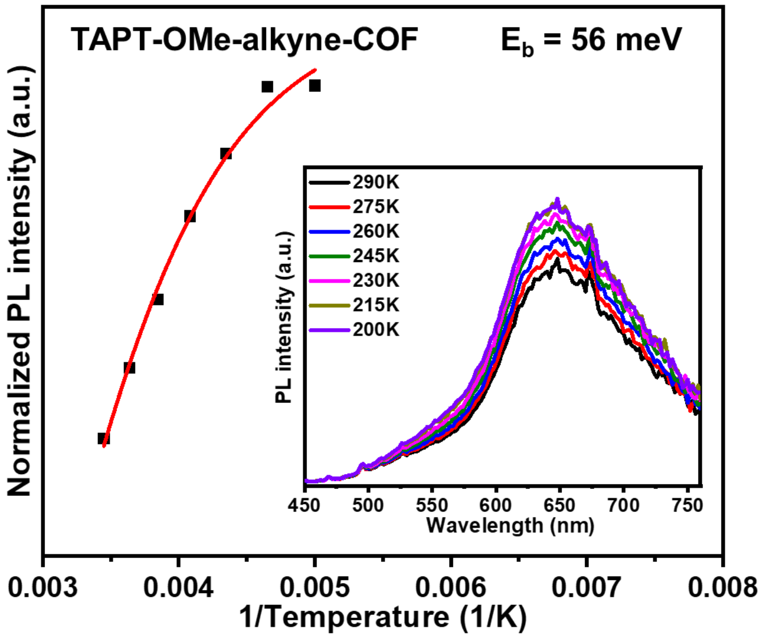


**Supplementary Figure 39 |** Integrated photoluminescence emission intensity as a function of temperature (Inset: temperature-dependent photoluminescence spectra, excitation at 400 nm) of TAPT-OMe-alkyne-COF. $E_b$ is derived from the Arrhenius equation.

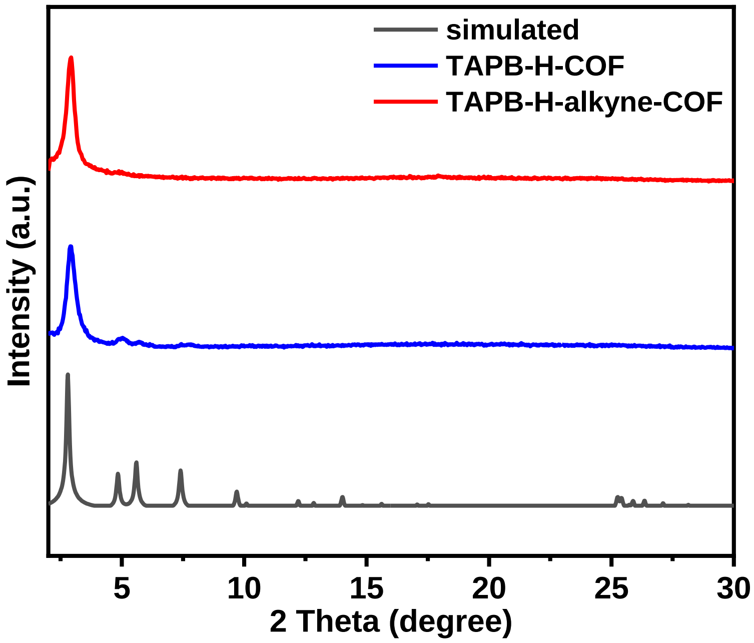


**Supplementary Figure 40 | Powder XRD patterns of TAPB-H-COF before and after post-modification.**

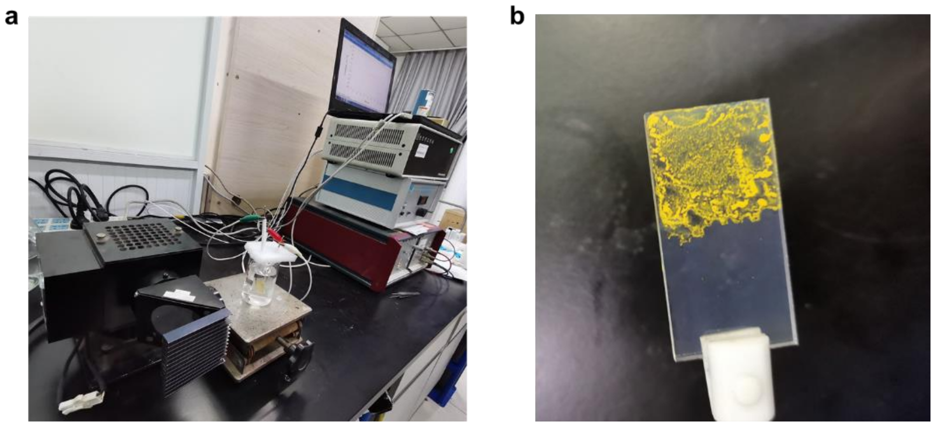


**Supplementary Figure 41 |** (a) Device setup for the photocurrent measurement. It contains the three parts (from left to right): xenon lamp; standard three-electrode system with the photocatalyst-coated FTO as a working electrode; the CHI 760E electrochemical workstation. The switch of the light is realized by a cardboard covered with tinfoil. (b) Photocatalyst-coated FTO (size: 20*40*2.2 mm; coated area: 20*20 mm).

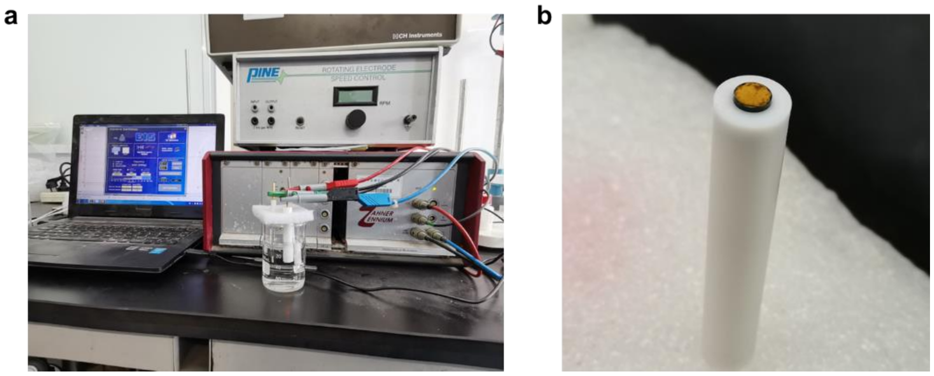


**Supplementary Figure 42 |** (a) Device setup for the EIS measurement. It contains the two parts (from near to far): standard three-electrode system with the photocatalyst-coated glassy carbon as the working electrode; the Zahner Zennium electrochemical workstation. (b) Photocatalyst-coated glassy carbon electrode (area: 0.2 $cm^2$).

## 3. Supplementary Tables 1-8

**Supplementary Table 1 | Theoretical calculation of the energy levels of different building blocks.**

| Building blocks | HOMO (eV) | LUMO (eV) | Band gap (eV) |
|---|---|---|---|
| **$TAPT_c$** | -8.22 | -1.16 | 7.06 |
| **$TAPB_c$** | -7.35 | -0.18 | 7.17 |
| **$Cl_c$** | -8.14 | 0.29 | 8.43 |
| **$H_c$** | -7.92 | 0.94 | 8.86 |
| **$OCCH_c$** | -7.57 | 0.27 | 7.84 |
| **$OH_c$** | -6.95 | 0.77 | 7.72 |
| **$OMe_c$** | -6.90 | 0.76 | 7.66 |

**Supplementary Table 2 | Calculated energy levels of different building blocks by another selection method for the fragments in the D-A pair.**

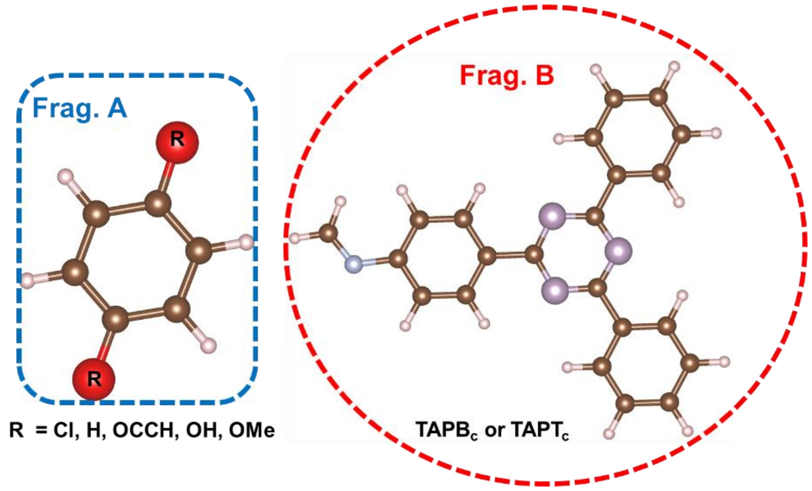


| Building blocks | HOMO (eV) | LUMO (eV) | Band gap (eV) |
|---|---|---|---|
| **Frag. B (TAPT$_c$)** | -7.97 | -1.39 | 6.58 |
| **Frag. B (TAPB$_c$)** | -7.32 | -0.40 | 6.92 |
| **Frag. A (Cl$_c$)** | -8.14 | 0.29 | 8.43 |
| **Frag. A (H$_c$)** | -7.92 | 0.94 | 8.86 |
| **Frag. A (OCCH$_c$)** | -7.57 | 0.27 | 7.84 |
| **Frag. A(OH$_c$)** | -6.95 | 0.77 | 7.72 |
| **Frag. A (OMe$_c$)** | -6.90 | 0.76 | 7.66 |

In comparison with that in Figure 1 in the text, the imine linkage (existed in the COF structure) is kept in the Fragment B (TAPT$_c$ or TAPB$_c$) in this selection method. The energy level of the Fragment B has been re-calculated. Based on the resulting energy levels, the D-A structure can be constructed between Frag. B (TAPT$_c$) and Frag.A (H$_c$, OCCH$_c$, OH$_c$ and OMe$_c$), or between Frag. B (TAPB$_c$) and Frag. A (OH$_c$ and OMe$_c$).

**Supplementary Table 3 | Excitation Characteristics (S and D) of the D-A pairs, derived from Multiwfn.**[a]

| Item | *D* (Å) | *S* | *S*/*D*(Å)$^{-1}$ |
|---|---|---|---|
| **TAPT-Cl** | 0.66 | 0.72 | 1.09 |
| **TAPT-H** | 0.78 | 0.74 | 0.95 |
| **TAPT-OCCH** | 1.19 | 0.72 | 0.61 |
| **TAPT-OH** | 1.66 | 0.69 | 0.42 |
| **TAPT-OMe** | 1.91 | 0.69 | 0.36 |
| **TAPB-OMe** | 0.30 | 0.63 | 2.10 |

[a]The S represents the calculated overlap of hole-electron distribution, and D represents the calculated distance between of hole and electron.

**Supplementary Table 4 | Excitation situation of the D-A pairs for $E_{opt}$.**[a]

| Item | $E_{opt}$ (eV) | Excited transition | Occupied orbital | Virtual orbital |
|---|---|---|---|---|
| **TAPT-Cl** | 3.94 | $S_0 \rightarrow S_1$ | HOMO | LUMO |
| **TAPT-H** | 3.96 | $S_0 \rightarrow S_1$ | HOMO | LUMO |
| **TAPT-OCCH** | 3.85 | $S_0 \rightarrow S_1$ | HOMO | LUMO |
| **TAPT-OH** | 3.52 | $S_0 \rightarrow S_1$ | HOMO | LUMO |
| **TAPT-OMe** | 3.46 | $S_0 \rightarrow S_1$ | HOMO | LUMO |
| **TAPB-OMe** | 3.83 | $S_0 \rightarrow S_1$ | HOMO | LUMO |

[a]The $E_{opt}$ represents the energy gap between $S_0$ and $S_1$.

**Supplementary Table 5 |** Calculated vertical ionization potential (IP), vertical electron affinity (EA), fundamental gap ($E_{fund}$), optical gap ($E_{opt}$) and exciton binding energy ($E_b$) of D-A pairs.

| Item | IP (eV) | EA (eV) | $E_{fund}$ (eV) | $E_{opt}$ (eV) | $E_b$ (eV) |
|---|---|---|---|---|---|
| **TAPT-Cl** | 6.79 | 2.55 | 4.24 | 3.94 | 0.30 |
| **TAPT-H** | 6.59 | 2.34 | 4.25 | 3.96 | 0.29 |
| **TAPT-OCCH** | 6.46 | 2.34 | 4.12 | 3.85 | 0.27 |
| **TAPT-OH** | 6.05 | 2.33 | 3.72 | 3.52 | 0.20 |
| **TAPT-OMe** | 5.96 | 2.34 | 3.62 | 3.46 | 0.16 |
| **TAPB-OMe** | 5.97 | 1.81 | 4.16 | 3.83 | 0.33 |

**Supplementary Table 6 | Actual Pt content loading to D-A COFs after photodeposition determined by ICP tests.**

| Sample | TAPT-OMe-COF | TAPT-H-COF | TAPT-Cl-COF | TAPB-OMe-COF |
|---|---|---|---|---|
| **content (wt%)** | 2.26 | 2.19 | 1.85 | 2.31 |

**Supplementary Table 7 | Actual Pt content loading to TAPB-OMe-alkyne-COF and TAPT-OMe-alkyne-COF after photodeposition determined by ICP tests.**

| Sample | TAPT-OMe-alkyne | TAPB-OMe-alkyne |
|---|---|---|
| **content (wt%)** | 1.82 | 1.78 |

**Supplementary Table 8 |** Excitation Characteristics (S and D) (derived from Multiwfn) of the D-A pairs after post-synthetic modification with phenylacetylene.[a]

| Item | D (Å) | S | S/D (Å$^{-1}$) |
| --- | --- | --- | --- |
| **TAPT-OMe** | 1.91 | 0.69 | 0.36 |
| **TAPB-OMe** | 0.30 | 0.63 | 2.10 |
| **TAPT-OMe-alkyne** | 2.90 | 0.63 | 0.22 |
| **TAPB-OMe-alkyne** | 2.23 | 0.61 | 0.27 |

[a]The S represents the calculated overlap of hole-electron distribution, and D represents the calculated distance between of hole and electron.

**Supplementary references**


1 Jeon, S., Park, S., Nam, J., Kang, Y. & Kim, J.-M. Creating patterned conjugated polymer images using water-compatible reactive inkjet printing. *ACS Appl. Mater. Interfaces* **8**, 1813-1818 (2016).

2 Tian, H. *et al.* Effect of different dye baths and dye-structures on the performance of dye-sensitized solar cells based on triphenylamine dyes. *J. Phys. Chem. C* **112**, 11023-11033 (2008).

3 Sun, Q. *et al.* Postsynthetically modified covalent organic frameworks for efficient and effective mercury removal. *J. Am. Chem. Soc.* **139**, 2786-2793 (2017).

4 Gomes, R., Bhanja, P. & Bhaumik, A. A triazine-based covalent organic polymer for efficient $CO_2$ adsorption. *Chem comm.* **51**, 10050-10053 (2015).

5 Bredas, J.-L. Mind the gap! *Mater. Horiz.* **1**, 17-19 (2014).

6 Wang, X. *et al.* Sulfone-containing covalent organic frameworks for photocatalytic hydrogen evolution from water. *Nat. Chem.* **10**, 1180-1189 (2018).

7 Guiglion, P., Butchosa, C. & Zwijnenburg, M. A. Polymer photocatalysts for water splitting: insights from computational modeling. *Macromol. Chem. Phys.* **217**, 344-353 (2016).

8 Lu, T. & Chen, F. Multiwfn: A multifunctional wavefunction analyzer. *J. Comput. Chem.* **33**, 580-592 (2012).

9 Humphrey, W., Dalke, A. & Schulten, K. VMD: visual molecular dynamics. *J. Mol. Graphics* **14**, 33-38 (1996).

10 Le Bahers, T., Adamo, C. & Ciofini, I. A. Qualitative index of spatial extent in charge-transfer excitations. *J. Chem. Theory Comput.* **7**, 2498-2506 (2011).

11 Li, X.-T. *et al.* Construction of covalent organic frameworks via three-component one-pot Strecker and Povarov reactions. *J. Am. Chem. Soc.* **142**, 6521-6526 (2020).
